# Supplementary material for: Vitamin D-mediated effects on airway innate immunity in vitro
Source: PLoS One. 2022 Jun 6;17(6):e0269647. doi: 10.1371/journal.pone.0269647 (PMC9170100; doi:10.1371/journal.pone.0269647)
Supplement: S1 Fig — Control (vehicle) media and S. aureus resulted in a slight decline of bacteria over 12 minutes, while bleach and S. aureus led to complete bacterial killing over the same time. (DOCX) [file pone.0269647.s001.docx]

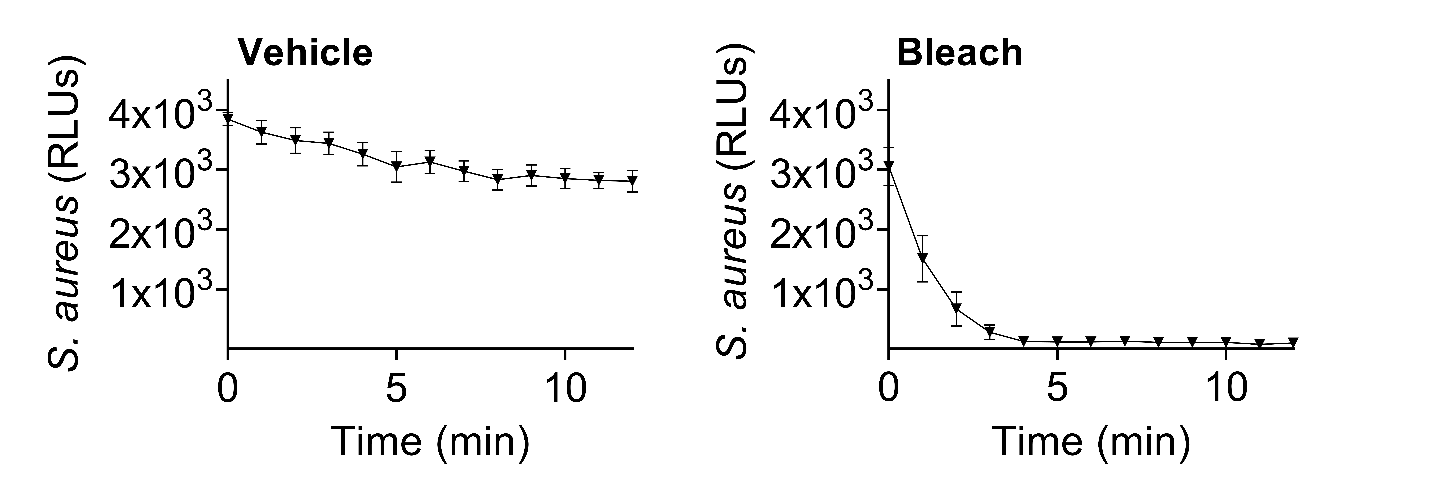


**S1 Fig.** **Relative light units of control conditions from S. aureus bacterial killing assays.** Control (vehicle) media and *S. aureus* resulted in a slight decline of bacteria over 12 minutes, while bleach and *S. aureus* led to complete bacterial killing over the same time.
