# Supplementary material for: Vitamin D-mediated effects on airway innate immunity in vitro
Source: PLoS One. 2022 Jun 6;17(6):e0269647. doi: 10.1371/journal.pone.0269647 (PMC9170100; doi:10.1371/journal.pone.0269647)
Supplement: S2 Fig — A clinically isolated strain of methicillin-resistant S. aureus was cultured overnight in tryptic soy broth (TSB), then sub-cultured (1:100) in TSB +/- 10−7 M calcitriol. Paired t-tests of % growth compared to time 0 (at hours 1–6) demonstrated no statistically significant differences between control and supplemented media (all p>0.05). This experiment was independent of airway mechanisms. (DOCX) [file pone.0269647.s002.docx]

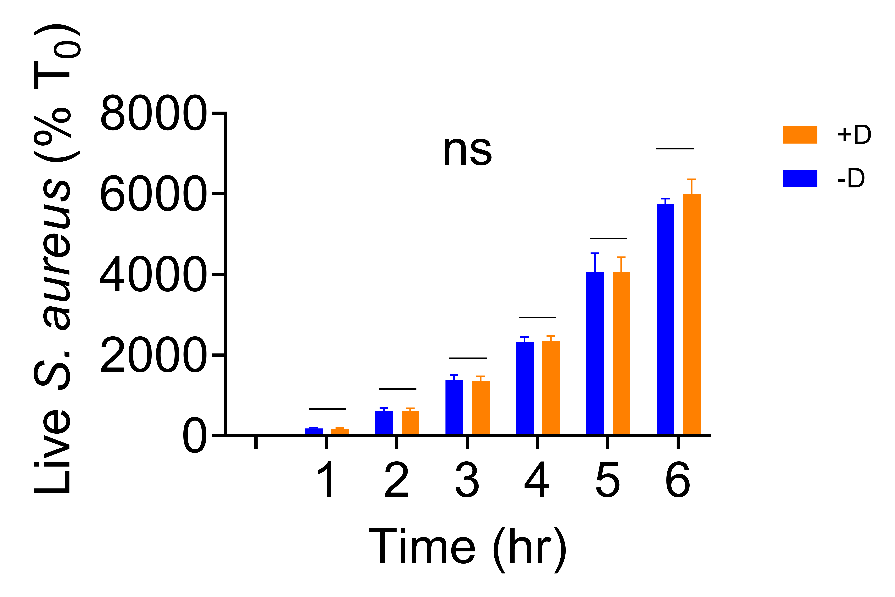


**S2 Fig.** ***S. aureus* growth in media with and without calcitriol supplementation.** A clinically isolated strain of methicillin-resistant *S. aureus* was cultured overnight in tryptic soy broth (TSB), then sub-cultured (1:100) in TSB +/- 10^-7^ M calcitriol. Paired t-tests of % growth compared to time 0 (at hours 1-6) demonstrated no statistically significant differences between control and supplemented media (all *p*>0.05). This experiment was independent of airway mechanisms.
